# Current Status of Long Non-Coding RNAs in Human Breast Cancer

**DOI:** 10.3390/ijms17091485

**Published:** 2016-09-06

**Authors:** Stefanie Cerk, Daniela Schwarzenbacher, Jan Basri Adiprasito, Michael Stotz, Georg C. Hutterer, Armin Gerger, Hui Ling, George Adrian Calin, Martin Pichler

**Affiliations:** 1 Division of Oncology, Department of Internal Medicine, Medical University of Graz, Graz 8026, Austria; stefanie.cerk@medunigraz.at (S.C.); daniela.schwarzenbacher@medunigraz.at (D.S.); jan.adiprasito@stud.medunigraz.at (J.B.A.); michael.stotz@medunigraz.at (M.S.); armin.gerger@medunigraz.at (A.G.); 2 Research Unit of Non-coding RNA and Genome Editing in Cancer, Medical University of Graz, Graz 8036, Austria; 3 Department of Urology, Medical University of Graz, Graz 8036, Austria; georg.hutterer@medunigraz.at; 4 Department of Experimental Therapeutics, The University of Texas MD Anderson Cancer Center, Houston, TX 77054, USA; hling@mdanderson.org (H.L.); gcalin@mdanderson.org (G.A.C.)

**Keywords:** breast cancer—carcinoma, long non-coding RNA, oncogene, tumor suppressor

## Abstract

Breast cancer represents a major health burden in Europe and North America, as recently published data report breast cancer as the second leading cause of cancer related death in women worldwide. Breast cancer is regarded as a highly heterogeneous disease in terms of clinical course and biological behavior and can be divided into several molecular subtypes, with different prognosis and treatment responses. The discovery of numerous non-coding RNAs has dramatically changed our understanding of cell biology, especially the pathophysiology of cancer. Long non-coding RNAs (lncRNAs) are non-protein-coding transcripts >200 nucleotides in length. Several studies have demonstrated their role as key regulators of gene expression, cell biology and carcinogenesis. Deregulated expression levels of lncRNAs have been observed in various types of cancers including breast cancer. lncRNAs are involved in cancer initiation, progression, and metastases. In this review, we summarize the recent literature to highlight the current status of this class of long non-coding lncRNAs in breast cancer.

## 1. Breast Cancer

According to the American Cancer Association, 1,685,210 new cancer cases and 595,690 cancer deaths are estimated to occur in the United States in 2016 [1]. Breast cancer is currently the most frequently diagnosed cancer in women, with an estimated 246,660 new cases—representing 29% of all cancer diagnoses in women- and moreover, 40,450 deaths in 2016 in the United States [1]. Breast cancer represents a highly heterogeneous disease in terms of clinical outcomes and biological behavior and thus can be classified with various methods into different subtypes [2]. In recent years, several novel molecular, cellular, tissue- and blood-based prognostic factors have been identified [3,4,5]. In routine clinical practice, however, relying on immunohistochemical analysis of the estrogen receptor (ER), progesterone receptor and her2/neu receptor, breast cancer is subdivided into hormone receptor positive, her2/neu receptor positive, and (lack of all three receptors) triple negative [6]. Though, the development of novel global transcriptome analysis enabled a further sub-classification into molecular subtypes, including luminal A, luminal B, HER2 enriched, claudin-low, basal-like, and normal breast like [6,7]. Moreover, Ki-67 can be used as a proliferation marker to distinguish between strongly endocrine responsive, low proliferative and good prognosis subtype, namely luminal A-like and less endocrine responsive, high proliferative, and poorer prognosis luminal B-like tumors [8].

All these different gene expression signatures are corresponding to classifiers of protein-coding genes but besides the molecular differentiation, each subtype is distinct, regarding clinical prognosis, sensitivity to drugs, as well as the response duration to treatment [9,10]. Novel concepts in genetics and molecular biology are leading to improved and higher resolution of the underlying events and increasing complexity in breast cancer biology [11,12].

## 2. Long Non-Coding RNAs (lncRNAs)

The protein-coding world represents only one side of the coin and, recently, tremendous efforts and progress have been made in identifying novel factors and concepts in breast cancer biology. Non-coding RNA molecules, which are by definition transcribed RNAs that never get translated into a protein (therefore named as ‘non-coding’), evolved as the new kids on the block in cell biology [13]. The Encyclopedia of DNA Elements (ENCODE) project has revealed that over 80% of the human genome is transcribed into RNAs with a biochemical function [14], while only about 1.5% of the human genome actually encodes for proteins [15]. In general, these non-coding RNAs (ncRNAs) represent a broad group of RNAs, including some of the classical ‘housekeeping’ RNAs, like transfer RNAs, ribosomal RNAs, small nucleolar RNAs, and small nuclear RNAs [14,16]. Basically, relying on transcript size, ncRNAs can be classified into two major groups: short ncRNAs, with less than 200 nucleotides (nt) and long ncRNAs (>200 nt). Former can be divided into at least four major classes: microRNAs (miRNAs), short interfering RNAs (siRNAs), transcription initiation RNAs (tiRNAs), and PipW-interacting RNAs (piRNAs) [17]. MicroRNAs in particular have been identified as important regulators of carcinogenesis in all types of cancer [18,19,20,21], including breast cancer stem cells [22], whereby they carry a promising potential to show up one day as diagnostic and prognostic cancer biomarkers [23]. lncRNAs, which represent the focus of this review, have received much attention due to their functional relevance in different physiological and pathological processes, as well as their tissue- and developmental-specific expression patterns [13,24,25,26]. lncRNAs outnumber protein-coding genes [27] and the total number of lncRNAs continue to rise due to improved RNA-sequencing, epigenomic technologies, and computational prediction techniques [28,29]. Most of lncRNAs are not highly conserved in their sequence [16,17,30] and generally show—compared to mRNAs—lower expression levels [26]. The majority of lncRNAs are transcribed by RNA polymerase II and undergo co-transcriptional and post-transcriptional processing events, like 5′-methylguanosine-capping, polyadenylation, splicing, and base modification [16,30,31]. Depending on their location with reference to protein-coding genes, lncRNAs can be classified into sense, antisense, intronic, and intergenic [16,32]. Predominantly, they are located in the nucleus, but nevertheless they are not strictly arranged in one particular compartment, rather, they can be found ubiquitously [26,33].

In general, lncRNAs depict a heterogeneous group of molecules that can be divided according to varying classification systems. One of this sub-classifications divides them into three categories, based on their functional characteristics: non-functional lncRNAs, lncRNAs for which the transcription-event alone is sufficient for their function but the transcript itself is not required, and functional lncRNAs that can act in a *cis* and/or in *trans* manner [30,31]. The latter is able to positively or negatively alter the expression or processing of their targets, which can be either coding or non-coding genes [13,34,35].

It is known that lncRNAs mediate several key cellular functions, like regulation of gene expression, genomic reprogramming, X-chromosome inactivation, genomic imprinting, nuclear compartmentalization, nuclear cytoplasmic trafficking, as well as RNA-splicing [16,36,37,38,39]. Due to their multiple and heterogeneous mechanisms of action, they characterize common ‘master regulators’ of gene expression, modulating it at epigenetic-, transcriptional-, as well as posttranscriptional levels [34,36,37,40,41,42,43,44,45].

Additionally, lncRNAs are also known to play a pivotal role in the control of the cell cycle and apoptosis; they are able to either act as tumor suppressor genes, whereas others are defined as oncogenes [46,47,48,49,50].

Genome wide associations studies (GWAS) in different cancer types revealed that over 80% of cancer-associated single nucleotide polymorphisms (SNPs) occur in non-coding regions of the genome. Only 3.3% of all cancer-related SNPs actually do change the protein amino acid sequence. The majority of SNPs is located in the introns of protein-coding genes (40%) or intergenic regions (44%), indicating an important role of these non-coding sequences in carcinogenesis [51]. Recently, numerous studies have demonstrated that lncRNAs are deregulated in cancer tissues and cells, suggesting that an aberrant expression might be an important contributor to tumorigenesis [49,52,53,54,55,56,57].

## 3. lncRNAs in Breast Cancer

As mentioned above, several studies were able to demonstrate that lncRNAs are frequently deregulated in various cancers. Meanwhile, numerous lncRNAs have been identified that show different expression patterns in breast cancer tissue compared to normal breast tissue [58,59,60]. For example, Yang and colleagues have identified more than 1300 lncRNAs that show significantly aberrant expression patterns in the HER-2-enriched subtype of breast cancer by using next generation sequencing [61]. Equally, Shen et al. [14] have figured out that over 1750 lncRNAs were differentially expressed in triple negative breast cancer (TNBC). These results clearly indicate that aberrant expression patterns of lncRNAs might play an important and often underestimated role in breast cancer carcinogenesis.

As already mentioned, lncRNA expression is much more cell-, tissue-, and developmental specific than those of mRNA. Hence, specific lncRNA expression patterns can be a useful tool to distinguish between the various breast cancer subtypes. Lv and colleagues have found differentially expressed lncRNAs to distinguish between TNBC and non-TNBC breast cancer. These lncRNAs may serve as individual diagnostic biomarkers and may be potential targets for individual therapy [10]. lncRNAs can be direct targets of ER in luminal A-like breast cancer cells and can serve as predictive biomarkers [62].Furthermore, Miano et al. [63] have found 133 ERα-dependent lncRNAs that are highly specific for luminal-like breast cancer and are therefore very promising in defining this specific subtype of breast cancer.

Another study has also provided a classification system of breast cancer using lncRNA expression [64]. They have found four lncRNA clusters that display different prognoses. The lncRNA HOX antisense intergenic RNA (HOTAIR) was significantly overexpressed in the HER2-enriched subgroup (cluster II), lncRNA HOTAIRM1 showed significantly higher expression in the basal-like subgroup (cluster I) and expression of estrogen receptor was associated with lncRNAs in cluster III and IV [64]. Two further studies have also established a TNBC classification system based on the expression profiles of both mRNAs and lncRNAs. It enables the division of TNBC into subtypes and determines subtype-specific lncRNAs that are potential biomarkers and molecular targets [65,66].

Jiang et al. [67] have developed an integrated mRNA-lncRNA signature that can classify TNBC patients into a low and a high risk group. The latter have higher risks of disease recurrence and achieve less benefit from taxane-based chemotherapy.

To summarize and detail the findings of previously published studies, we are focusing in the following paragraphs on the so far best characterized candidates (Table 1).

## 4. lncRNA Candidates in Breast Cancer

### 4.1. H19

The *H19* gene was one of the first discovered genes that was recognized to be submitted to genomic imprinting. It is located in the 11p15.5 region within 200 kilobase (kb) downstream of the Insulin-like growth factor 2 (*IGF-2*) gene [68]. These two genes are oppositely imprinted, *IGF-2* is transcribed from the paternal allele [69], while *H19* is only maternally expressed [70]. The *H19* gene is transcribed by RNA polymerase II and encodes a 2.3 kb lncRNA. The transcript is spliced, polyadenylated, capped, and translocated into the cytoplasm and it is associated with polysomes [71,72,73,74]. H19 expression is developmentally regulated and it is highly expressed in extraembryonic tissue (placenta), as well as embryo proper and fetal tissue. After birth, its expression is repressed except in a few adult tissues, including mammary and adrenal gland, as well as in the uterus [72,73]. H19 upregulation has been reported in various cancers, such as bladder-, lung-, esophageal-, cervical-, and breast cancer [74,75,76,77,78,79,80], whereby it is often associated with poor prognosis [81,82,83]. It regulates genes involved in metastasis and blood vessel development, suggesting an important role in tumor invasion and angiogenesis [72,84]. Further, H19 operates as an oncogene through different mechanisms. It can function as a *Myc*-upregulated gene to potentiate tumorigenesis in several cell types, including breast cancer cells [85]. It also has been reported that H19 acts as a molecular sponge to regulate members of the let-7 miRNA family, which all play important roles in development, metabolism, and cancer [86]. Moreover, Cai and Cullen have described that H19 is able to function as a precursor of *miR-675*, suggesting that it might act as a gene-expression regulator at the post-transcriptional level [87].

On top of that, H19 RNA is also involved in epithelial to mesenchymal transition (EMT), as well as in mesenchymal to epithelial transition (MET). It has been shown that numerous EMT inducers lead to increased H19/*miR-675* expression. Furthermore, H19 RNA suppresses the expression of the E-cadherin protein and is essential for upregulation of the EMT related transcription factor Slug by Transforming Growth Factor β (TGF-β). In contrast, it was also reported that *miR-675* might represent a MET promoter by downregulating the EMT key mediator Twist-related protein 1 (Twist1) [84,88,89]. In breast cancer, H19 overexpression promotes tumor progression [90] and leads to increased cell proliferation due to promoting the G-S transition through positive control by the transcription factor E2F1 [72].

Adriaenssens et al. [79] have reported that an increase of *H19* gene expression can be observed in breast cancer compared to healthy tissues and that upregulation of the *H19* gene is correlated with the tumor grades and the presence of estrogen- as well as with progesterone receptors.

Vennin and colleagues have shown that an overexpression of H19/*miR-675* enhances breast cancer cell aggressiveness, including an increased proliferation and migration rate in vitro, and increases tumor growth and metastasis in vivo. Additionally, they identified the ubiquitin ligase E3 family (*c-CbI* and *CbI-b*) as direct targets of *miR-675* in breast cancer cells, providing novel mechanistic insights into a role of lncRNA H19 in breast cancer development [73].

### 4.2. HOTAIR

In 2007, Rinn et al. [91] have identified HOX antisense intergenic RNA (*HOTAIR*) that is located on chromosome 12q13.13 in the HOXC locus. It depicts a trans-acting, spliced, and polyadenylated lncRNA that is 2.2 kb in length and is considered an oncogene [91,92,93].

HOTAIR functions as molecular scaffold and interacts with the methyltransferase Polycomb Repressive Complex 2 (PRC2). It regulates chromosome occupancy by enhancer of zeste homolog 2 (EZH2 -a subunit of PRC2), resulting in histone H3 lysine-27 trimethylation of the HOXD locus [91,92,93]. Furthermore, HOTAIR recruits PRC2 to specific target genes genome-wide, leading to histone H3 lysine-27 trimethylation and epigenetic silencing of metastasis suppressor genes [93,94,95]. Moreover, HOTAIR is able to interact with the Lysine-specific demethylase 1 (LSD1) complex, leading to epigenetic gene silencing due to demethylation of histone H3 at lysine 4 [96,97]. Through these functions, HOTAIR seems to affect the gene expression of several genes involved in various cellular functions [94]. In general, HOTAIR is ubiquitously overexpressed in most human cancers and correlated with tumor invasion, progression, metastasis, and poor prognosis [91,93]. Its pervasive deregulation in cancers was first observed in breast cancer. Gupta and colleagues have reported higher HOTAIR expression levels in primary breast tumors compared with adjacent tissue [94]. Estradiol transcriptionally induces HOTAIR via functional estrogen response elements within the promoter region, which might contribute to breast cancer progression [98,99]. Tao and colleagues have recently demonstrated that estradiol induces HOTAIR expression via G-protein-coupled estrogen receptor-1 mediated *miR-148a* inhibition in breast cancer [100]. Padua et al. [101] have showed that HOTAIR overexpression upregulates the genes related to EMT. In addition, they observed elevated HOTAIR levels in a colon cancer stem cell subpopulation compared with a non-stem cell subpopulation, indicating that HOTAIR might be required for EMT and stemness maintenance.

Particularly in breast cancer, HOTAIR is able to indirectly downregulate *miR-7* via HoxD10 inhibition, resulting in EMT progression. *miR-7* represents a metastasis-suppressing miRNA that can reverse EMT by downregulating the signal transducer and activator of transcription 3 (STAT3) pathway in breast cancer cells [102].

### 4.3. MALAT-1

The metastasis-associated lung adenocarcinoma transcript 1 (*MALAT-1*) is located on chromosome 11q13 and encodes an 8 kb lncRNA. MALAT-1 defines one of the first ever known ncRNAs that are associated with lung cancer. Since its discovery, several studies have reported the link of MALAT-1 to other cancers, including gallbladder-, cervical-, non-small cell lung- (NSCLC), colorectal-, and breast cancer. In most cases, MALAT-1 is upregulated in cancer and is associated with metastasis, cell proliferation, apoptosis, and migration, as well as with clinically unfavorable prognostic parameters [16,49,103,104,105,106].

MALAT-1 activates the MAPK extracellular signal-regulated kinase/mitogen-activated protein kinase (ERK/MAPK) pathway, resulting in an increased proliferation of gallbladder cancer cells [106]. Another mechanistic study has demonstrated that MALAT-1 induces the expression of the oncogenic transcription factor myb-related protein B (B-MYB) that is required for the transcription of various genes involved in mitotic progression [107]. Furthermore, Ying and colleagues have reported that MALAT-1 might lead to bladder cancer cell migration due to the promotion of EMT by activating Wnt signaling [108].

A recent study performed by Xu et al. [109] was able to demonstrate that MALAT-1 is downregulated in breast tumor cell lines as well as in breast cancer tissue. MALAT-1 regulates metastasis in breast cancer by inducing EMT via activation of the phosphatidylinositide 3-kinase—protein kinase B (PI3K-AKT) pathway.

Wang et al. [110] have recently demonstrated that MALAT-1 is linked to overall survival, recurrence free survival, and death-free survival, however, association between MALAT-1 levels and clinical features like TNM stage, lymph node metastasis, and distant metastasis were diverse in different types of cancer. Therefore, MALAT-1 may serve as a biomarker, but its value differs in various cancers.

In addition, another study has not found a link between MALAT-1 overexpression TNM grade, lymph node status and tumor size, indicating that high levels of MALAT-1 do not have a major role in the aggressive behavior of breast carcinoma [111]. The same group has found an alternative splice variant, namely Δsv-MALAT-1 that show very different expression pattern relative to full length MALAT-1. Δsv-MALAT-1 was mainly downregulated in breast tumors, therefore it can serve as an independent prognostic marker. Its expression was associated with alterations of the pre-mRNA alternative splicing machinery, the Drosha-DGCR8 complex that is required for ncRNA biogenesis as well as with an activation of the PI3K-AKT pathway [111].

In TNBC and HER2+ breast cancer, MALAT-1 expression could be used as a potential prognostic marker, but not in luminal patients. In TNBC and HER2+ subtypes, MALAT-1 level could be used to predict tumor recurrence and metastasis in lymph-node negative patients [112].

### 4.4. XIST

The X inactive-specific transcript (XIST) is an 17 kb spliced and polyadenylated lncRNA [113] encoded by the *XIST* gene, which is located in the X-inactivation center on the inactive X-chromosome [114]. XIST represents the key player of X-chromosome silencing in female cells. It is typically expressed by all female somatic cells, but its expression has been found to be lost in female breast-, ovarian-, as well as cervical cancer cell lines. Typically, only one active X-chromosome is present in human cells, but in human cancers, a loss of a normal X-chromosome can be observed along with dysregulated XIST expression [114,115]. Cancer cells have shown either downregulation or upregulation of XIST, suggesting its complex and controversial role in cancer biology [114,116].

### 4.5. GAS5

The Growth arrest-specific 5 (*GAS5*) gene is located on chromosome 1q25.1 and encodes small nucleolar RNAs, microRNAs, and PIWI-interacting RNAs, in addition to lncRNA. It consists of 12 exons, which are alternatively spliced into two possible mature lncRNAs [117]. GAS5 lncRNA accumulates in growth arrested cells due to interaction with the mechanistic Target of Rapamycin (mTOR) pathway and through nonsense mediated decay [118].

The involvement of GAS5 in human cancers was first studied in breast cancer. In 2009, Mourtada-Maarabouni and colleagues reported that GAS5 is downregulated in breast cancer tissues [119]. Generally, GAS5 is found to be downregulated in various cancers and low expression levels are often predictive of poor prognosis in cancer patients [50,117,119,120,121]. Moreover, GAS5 promotes cell proliferation and /or apoptosis in different cell types, including breast cancer cells and its tumor suppressor role is indicated by its inhibition of breast tumor growth [117,122].

Li et al. [123] have found that GAS5 levels are decreased in trastuzumab-resistant SKBR-3/Tr cells and in breast cancer tissue from trastuzumab-treated patients. GAS5 knockdown increased cell proliferation and tumor growth in vivo and low levels of GAS5 correlated with histological grade and advanced TNM stage. That indicates that GAS5 is reduced by trastuzumab and may act as a tumor suppressor in trastuzumab-resistant breast cancer.

### 4.6. PANDAR

*PANDAR* (promoter of CDKN1A antisense DNA damage activated RNA) is located approximately 5 kb upstream of the cell cycle gene Cyclin-Dependent Kinase Inhibitor 1A (CDKN1A) transcription start site and encodes for a 1.5 kb lncRNA. Upon DNA damage, expression of PANDAR is induced by p53. PANDAR interacts with the transcription factor NF-YA to restrict the expression of pro-apoptotic genes and enables cell cycle arrest. Silencing of PANDAR increases the DNA damage-induced apoptosis and PANDAR has been found to control the entry and exit into and out of senescence [54,124,125,126]. Han and colleagues have reported that PANDAR is downregulated in NSCLC and low expression levels are associated with poor prognosis [127].

Several controversially discussed studies have demonstrated that PANDAR is upregulated in hepatocellular carcinoma, gastric cancer, and breast cancer [54,128,129], indicating its potentially complex role in cancer. As mentioned, PANDAR is upregulated in breast cancer tissues, as well as in breast cancer cell lines and functions as a tumor-promoting lncRNA by regulating G1/S transition. PANDAR-mediated G1/S transition and promotion of cell growth is, at least in parts, a result of the suppression of its downstream target p16^INK4A^ [54].

### 4.7. CCAT1

Colon cancer-associated transcript 1 (CCAT1) is a 2.6 kb transcript [130]. CCAT1 has been found to be upregulated in many cancers, including gastric-, hepatocellular-, gallbladder-, colorectal-, colon-, and breast cancer [52,130,131,132,133,134,135]. Its increased expression is associated with clinical stage, lymph node metastases, and survival after surgery in colon cancer patients [130], as well as with poor prognosis in hepatocellular carcinoma patients [134].

Ma and colleagues have reported that CCAT1 upregulates the *miRNA-218-5p* target gene B cell-specific Moloney murine leukemia virus integration site 1 (*Bmi1*) by acting as *miRNA-218-5p* sponge, resulting in an increased proliferation and invasiveness of gallbladder cancer cells [135]. Upregulation of CCAT1 promotes proliferation and migration of hepatocellular carcinoma cells, due to its function as let-7 sponge [134].

A factor potentially contributing to the deregulation of CCAT1, is c-Myc, an important regulator of cell cycles, proliferation, differentiation, and apoptosis that is also known as the oncogene [131,136]. c-Myc might promote CCAT1 transcription by directly binding to its promoter region, resulting in an upregulation of CCAT1 expression [130,137]. Upregulated CCAT1 expression correlates with aggressive disease progression and poor prognosis in breast cancer patients, albeit the detailed functional mechanisms in breast cancer are still unknown [52].

### 4.8. CCAT2

Colon cancer-associated transcript 2 (CCAT2) represents a novel lncRNA located at the highly conserved 8q24.21 region [138]. The group around George A. Calins lab was able to demonstrate that an elevated CCAT2 expression is associated with the development of metastases and poor prognosis within various cancers, including breast cancer [53,139,140,141,142,143].

Cai et al. [139] have figured out that CCAT2 is upregulated in breast cancer tissues compared to adjacent non-tumor tissues and that its expression is correlated with clinico-pathological prognostic factors. Patients with an elevated CCAT2 expression had a significantly poorer prognosis than those with low expression levels. Moreover, the relative CCAT2 expression level correlated with the overall survival rate of breast cancer patients.

Suppressing CCAT2 expression by siRNAs leads to a decreased cell proliferation and invasion in vitro and inhibits tumorigenesis in vivo. A knockdown of CCAT2 affects the Wnt/β-catenin signaling pathway, by suppressing β-catenin activity, whose activation leads to the development of human cancers, including breast cancer [139]. Taken together, these findings indicate that an elevated CCAT2 expression is associated with the progression and development of breast cancer.

Redis et al. [142] have identified the tissue- and subcellular localization of CCAT2 in breast cancer by in situ hybridization, using 16 non-tumor- and 18 tumor samples. CCAT2 appeared to have higher expression levels in the epithelial component of breast cancer tissues compared with those of non-tumor tissues. CCAT2 was detected in the nucleus as well as in the cytoplasm, with a more intense staining within the nuclear compartment, indicating enrichment within this compartment. The same group of authors have recently proposed different metabolic pathway activation patterns depending on a single nucleotide polymorphism, which influences the 3-dimensional structure of CCAT2, indicating the well-known conformational importance of lncRNA functions [144].

### 4.9. UCA1

The Urothelial carcinoma associated 1 (*UCA1*) gene is located on the positive strand of chromosome 19p13.12. The sequence consists of three exons and at least three splice variants do exist that are 1.4, 2.2, and 2.7 kb, respectively in length, whereof the 1.4 kb transcript is the most abundant one [145,146].

The *UCA1* gene encodes for an lncRNA that is highly expressed in various carcinomas including bladder-, colorectal- and breast cancer [145,147,148,149], suggesting that UCA1 might serve as a potential biomarker for diagnostic purposes in the future [146].

Moreover, UCA1 acts as a microRNA sponge, which has been demonstrated by Nie and colleagues [150]. The authors have pointed out that a UCA1 upregulation promotes the proliferation of NSCLC cells partly through acting as an *miR-193a-3p*-sponge [150]. Na and colleagues have reported that lncRNA UCA1 regulates the proliferation in prostate cancer cells through regulation of kruppel-like factor 4 (*KLF4*) and keratin 6/13. UCA1 loss-of-function experiments inhibited cell proliferation and induced apoptosis, at least partly, through inactivation of the KLF4-KRT6/13 signalling pathway [151].

In breast cancer, UCA1 modulates cell growth and apoptosis, at least partially by interacting with miR-143, a microRNA with a tumor suppressive role in breast cancer [147] and colorectal cancer [152]. Through direct binding, UCA1 lowers the expression levels and reduces the biological effects of miR-143 [147]. Huang et al. [153] have demonstrated the oncogenic role of UCA1 in breast cancer, by showing that UCA1 promotes cell growth in vitro and in vivo. Their study suggests that UCA1 suppresses the tumor suppressor p27 through interaction with heterogeneous nuclear ribonucleoproteins I (hnRNP I).

### 4.10. EPB41L4A-AS2

The EPB41L4A-AS2 gene is located on chromosome 5p22.2 and encodes for a recently identified antisense lncRNA of unknown function. Xu et al. [154] have demonstrated that overexpression of EPB41L4A-AS2 inhibits tumor cell growth in renal-, lung-, and breast cancer cell lines, suggesting that it might act as a tumor suppressor by mediating cell proliferation. Downregulation of EPB41L4A-AS2 is linked with poor survival in these three cancers, indicating that its downregulation might contribute to tumorigenesis, as well as disease progression.

In breast cancer, EPB41L4A-AS2 is associated with tumorigenesis and chemoresistance, and it seems to be involved in the estrogen synthesis regulation. Furthermore, the expression pattern differs with tumor grade, tumor size, disease stage, receptor status, and molecular subtype, indicating that different molecular subtypes are more or less associated with this lincRNA [154].

### 4.11. BC040587

BC040587 depicts a novel lncRNA located on chromosome 3q13.31. The expression level of BC040587 in breast cancer tissue, as well as in breast cancer cell lines are low in comparison to normal tissues and cell lines, respectively, indicating its potential function as a tumor suppressor. Patients with a low BC040587 expression pattern tend to have a higher risk of poor grade of tumor differentiation. Moreover, overall survival was significantly decreased in patients with a low BC040587 expression compared to those with an elevated expression pattern, indicating its potential clinical relevance [155].

### 4.12. SPRY4-IT1

SPRY4 intronic transcript 1 (*SPRY4-IT1*) is 708 bp in length and located on chromosome 5 [156]. Several studies were able to demonstrate that SPRY4-IT1 promotes cell growth, invasion and inhibits apoptosis in several types of cancer, including breast cancer [156,157,158,159,160]. Recently, Ru and colleagues have reported that SPRY4-IT1 promotes EMT, invasion, and migration through association with Snail1 in osteosarcoma cells [160]. Snail1 is a transcription factor of the C2H2-type zinc-finger family that induces EMT through silencing of the E-cadherin expression [161]. Shi et al. have reported that an increased expression of SPRY4-IT1 is associated with a larger tumor size and later stage of tumor development in breast cancer patients and that a SPRY4-IT1 knockdown leads to suppressed cell proliferation and induced apoptosis in breast cancer cells. Additionally, the authors identified *ZNF703* as a downstream target gene and demonstrated that *ZNF703* promotes proliferation and suppresses apoptosis in vivo [156].

### 4.13. NBAT-1

lncRNA neuroblastoma associated transcript-1 (NBAT-1) is downregulated in numerous types of cancer, including breast cancer, which suggests a potential function as tumor suppressor [48,162,163]. A low expression of NBAT-1 increases cell migration and invasion and is correlated with poor prognosis in neuroblastoma, as well as in clear cell renal cell carcinoma patients [48,163]. A mechanistic study was able to demonstrate that NBAT-1 acts as a tumor suppressor by interacting with EZH2, a subunit of the global gene expression regulator PRC2 complex [163]. EZH2 expression is associated with increased tumor cell proliferation and regulates cell invasion mostly through mediating transcriptional silencing of tumor suppressor genes like E-cadherin [164,165,166].

In breast cancer, low NBAT-1 expression levels are associated with poor survival, as well as the development of lymph node metastases. Hu et al. [162] have showed that NBAT-1 upregulates Dickkopf WNT signaling pathway inhibitor 1 (DKK1), through interacting with EZH2. DKK1 represents an inhibitor of the WNT signaling pathway and might be responsible for NBAT-1’s effect in suppressing migration and invasion of breast cancer cells.

### 4.14. AK058003

lncRNA AK058003 is an 1197 bp long transcript located on the forward strand on locus 10q22 [167]. The expression levels have been shown to be elevated in breast cancer tissues and its upregulation promotes proliferation, invasion, and metastasis by regulating the γ-*synuclein* gene (*SNCG*) expression. *SNCG* (also known as breast cancer-specific protein 1) encodes a synaptic protein and its expression is closely associated with tumor invasion and metastasis in various cancers, including esophageal-, stomach-, liver-, and breast cancer, as well as with clinical stage and lymph node metastases [168,169].

### 4.15. Z38

The newly discovered lncRNA Z38 is highly expressed in breast cancer cells and breast cancer tissue. A knock-down of Z38 expression using siRNAs suppresses cell proliferation, tumor growth and tumorigenesis, and induces apoptosis. However, detailed functional mechanisms involved in the regulatory roles of Z38 in cancers are still unknown [47].

### 4.16. FGF14-AS2

A recently published study conducted by Yang et al. [170] has demonstrated that lncRNA FGF14-AS2, which is an anti-sense transcript for FGF14, is significantly downregulated in breast cancer tissue compared with adjacent normal tissue. Furthermore, the expression of FGF14-AS2 has been shown to be negatively correlated with tumor size, lymph node metastases, and clinical stage in two independent cohorts, indicating that it might act as a tumor suppressor gene. Specifically, this finding was confirmed when integrating FGF14-AS2 into multivariate analyses [170]. However, due to the lack of experimental data, an interpretation of these findings is difficult and not connected to an appropriate biological context yet.

### 4.17. MVIH

lncRNA associated with microvascular invasion in hepatocellular carcinoma (MVIH) has been found to be upregulated in hepatocellular carcinoma (HCC) [171], non-squamous cell lung cancer (NSCLC) [172] and breast cancer cells [173]. In HCC, it promotes tumor growth and intrahepatic metastases by activating angiogenesis [171]. In NSCLC, MVIH promotes cell proliferation and invasion of cancer cells. Moreover, the MVIH expression levels correlate with TNM stages, tumor size, lymph node metastasis, and poor prognosis [172]. In breast cancer, elevated expression levels of MVIH influence cell proliferation, apoptosis, and the cell cycle, whereby it correlates with high Ki67 staining, poor overall- and disease-free survival [173].

### 4.18. LINK-A

Long-Intergenic Noncoding RNA for Kinase Activation (LINK-A) is a 1540 bp transcript that is located in the cytoplasm. Basal-like breast cancer exhibits significantly elevated LINK-A expression relative to HER2+, luminal A-like, luminal B-like, and normal-like subtypes. Furthermore, *LINK-A* correlates with unfavorable recurrence free survival for breast cancer patients.

Mechanistically, LINK-A is critical for growth factor-induced normoxic Hypoxia-Inducible Factor α (HIF1α) signaling pathway. It is required for a Heparin-binding-epidermal-growth-factor-like growth factor (HB-EGF)-triggered, Epidermal Growth Factor Receptor (EGFR): glycoprotein non-metastatic b (GPNMB) heterodimer-mediated recruitment of breast tumor kinase (BRK) to GPNMB. This results in enzymatic activation of BRK that then, together with Leucinreicher-Repeat-Serin/Threoninkinase 2 (LRRK2), is also recruited by LINK-A, phosphorylate HIF1α at Tyr565, and Ser797, respectively. The Tyr565 phosphorylation interferes with Pro564 hydroxylation, resulting to normoxic HIF1α stabilization, while phosphorylation of Ser797 enables HIF1α-p300 interaction, leading to activation of HIF1α target genes upon HB-EGF stimulation. LINK-A expression as well as the activation of LINK-A-dependent HIF1α signaling pathway correlate with TNBC, promote breast cancer glycolysis reprogramming and tumorigenesis. Therefore, LINK-A may serve as potential target to block a normoxic HIF1α signaling pathway in TNBC [174].

### 4.19. DSCAM-AS1

DSCAM-AS1 is an estrogen receptor α (ERα)-dependent lncRNA and is antisense intronic within the *DSCAM *(Down Syndrome Cell Adhesion Molecule) gene. It highly specific for luminal breast cancer and can therefore be used as biomarkers of this subtype. DSCAM-AS1 knockdown mimics some features of ERα silencing, like reduction of cellular growth, increase of apoptosis, and induction of EMT markers without influencing ERα expression. To understand the mechanistic function of DSCAM-AS1, further studies are needed [63].

## 5. Summary

Within a relatively short period of time, lncRNAs have become recognized as a significant part of the eukaryotic transcriptome. They obviously play a crucial role in diverse important cellular processes, like epigenetic regulation, chromatin remodeling and splicing, whereby their dysregulation is able to contribute to the development and progression of cancer. lncRNAs are acting via manifold mechanisms and a detailed characterization of their modes of action will allow their use as potential biomarkers for diagnostic, as well as therapeutic purposes in the treatment of cancer patients in the future.

## Figures and Tables

**Table 1:** Known lncRNAs in breast cancer. This list is demonstrating their location and main function.

Name	Location	Tumor Suppressor/Oncogene	Function
H19	11p15.5	oncogene	miRNA sponge miRNA precursor
HOTAIR	12q13.13	oncogene	molecular scaffold; epigenetic gene silencing
MALAT-1	chromosome 11q13	oncogene	activates ERK/MAPK pathway; induces expression of *B-MYB*; promotes EMT by activating Wnt signaling
XIST	inactive X-chromosome		X-chromosome silencing
GAS5	1q25.1	tumor suppressor	interaction with the mTOR pathway
PANDAR	~5 kb upstream of *CDKN1A*		regulation of G1/S transition
CCAT1		oncogene	miRNA sponge
CCAT2	8q24.21	oncogene	regulation of Wnt/β-catenin signaling pathway
UCA1	19p13.12	oncogene	microRNA sponge; regulation of KLF4-KRT6/13 signalling pathway
EPB41L4A-AS2	5p22.2	tumor suppressor	unknown
BC040587	3q13.31	tumor suppressor	unknown
SPRY4-IT1	chromosome 5	oncogene	
NBAT-1		tumor suppressor	mediating transcriptional silencing
AK058003	10q22	oncogene	regulating γ-*synuclein* gene (*SNCG*) expression
Z38		oncogene	unknown
FGF14-AS2		tumor suppressor	unknown
MVIH		oncogene	unknown
LINK-A		oncogene	Rrgulation of HIF1α signaling pathway
DSCAM-AS1		oncogene	unknown

## Abbreviations

Bmi1	B cell-specific Moloney murine leukemia virus integration site 1
B-MYB	myb-related protein B
BRK	breast tumor kinase
*CCAT1*	Colon cancer-associated transcript 1
*CCAT2*	Colon cancer-associated transcript 2
*CDKN1A*	Cyclin-Dependent Kinase Inhibitor 1A
DKK1	dickkopf WNT signaling pathway inhibitor 1
*DSCAM*	Down Syndrome Cell Adhesion Molecule
EGFR	Epidermal Growth Factor Receptor
EMT	epithelial to mesenchymal transition
ER	estrogen receptor
ERK/MAPK pathway	MAPK extracellular signal-regulated kinase/mitogen-activated protein kinase pathway
ERα	estrogen receptor α
EZH2	enhancer of zeste homolog 2
GAS5	Growth arrest-specific 5
GPNMB	Glycoprotein non-metastatic b
HB-EGF	Heparin-binding-epidermal-growth-factor-like growth factor HCC: hepatocellular carcinoma
HER2	human epidermal growth factor receptor 2
HIF1α	Hypoxia-Inducible Factor α
hnRNP I	Heterogeneous nuclear ribonucleoproteins I
HOTAIR	HOX antisense intergenic RNA
IGF-2	Insulin-like growth factor 2
KLF4	Kruppel-like factor 4
LINK-A	Long-Intergenic Noncoding RNA for Kinase Activation
LRRK2	Leucinreicher-Repeat-Serin/Threoninkinase 2
LSD1	Lysine-specific demethylase 1
MALAT-1	metastasis-associated lung adenocarcinoma transcript 1
ME	mesenchymal to epithelial transition
miRNA	microRNAs
mTOR	mechanistic Target of Rapamycin
MVIH	lncRNA associated with microvascular invasion in hepatocellular carcinoma
NBAT-1	neuroblastoma associated transcript-1
ncRNA	non-coding RNA
NSCLC	non-small cell lung cancer
PANDAR	promoter of *CDKN1A* antisense DNA damage activated RNA
PI3K-AKT	phosphatidylinositide 3-kinase—protein kinase
PR	progesterone receptor
PRC2	Polycomb Repressive Complex 2
SNCG	γ-synuclein gene
SNP	single nucleotide polymorphisms
SPRY4-IT1	SPRY4 intronic transcript 1
STAT3	Signal transducer and activator of transcription 3
*TGF*-β	Transforming Growth Factor β
TNBC	triple negative breast cancer
*Twist1*	Twist-related protein 1
UCA1	Urothelial carcinoma associated 1
XIST	X inactive-specific transcript
